# Rickettsial Meningoencephalitis With Multiorgan Failure in a Previously Healthy Young Adult

**DOI:** 10.7759/cureus.111032

**Published:** 2026-06-17

**Authors:** Carolina Pico Mendoza, Rubi De la Rosa Lopez, Luisa F Vazquez Enriquez, Monica Berumen Rodriguez, Kenia Dominguez Romo

**Affiliations:** 1 Internal Medicine, Hospital General de Zona No. 33, Instituto Mexicano del Seguro Social, Monterrey, MEX

**Keywords:** central nervous system involvement, doxycycline, encephalitis, inoculation eschar, multiorgan failure, rickettsial meningoencephalitis, rickettsiosis

## Abstract

Rickettsioses are zoonotic infections caused by obligate intracellular gram-negative bacteria of the genus *Rickettsia*. Although they commonly present as an acute febrile illness with rash, central nervous system involvement is uncommon and may be associated with severe morbidity and mortality. A previously healthy 28-year-old man presented with fever and acute gastrointestinal symptoms followed by the development of a centrifugal macular rash involving the trunk, extremities, palms, and soles. He subsequently developed progressive somnolence and language disturbances, progressing to severe neurological impairment requiring mechanical ventilation. Laboratory studies revealed thrombocytopenia, acute kidney injury, hyponatremia, and hepatic dysfunction. Brain computed tomography (CT) demonstrated subtle loss of gray-white matter differentiation and mild sulcal effacement, findings suggestive of diffuse cerebral edema. Cerebrospinal fluid analysis, blood cultures, and multiplex polymerase chain reaction (PCR) testing for common bacterial, viral, and fungal pathogens were unrevealing. Further examination identified an inoculation eschar on the left lower extremity. Conventional serum PCR testing was positive for *Rickettsia *spp., supporting the diagnosis of rickettsial meningoencephalitis, although species-level identification was not available. Intravenous doxycycline was initiated in addition to empiric antimicrobial therapy; however, the patient developed progressive multiorgan failure and failed to demonstrate meaningful neurological recovery. This case highlights the importance of considering rickettsiosis in the differential diagnosis of meningoencephalitis in endemic regions, particularly when fever, rash, and neurological manifestations coexist. Early recognition and prompt initiation of doxycycline remain essential to reduce the risk of severe neurological complications and poor clinical outcomes.

## Introduction

Rickettsioses are zoonotic infections caused by obligate intracellular gram-negative bacteria belonging to the family Rickettsiaceae, which includes the genera *Rickettsia *and *Orientia*. Members of the genus *Rickettsia *are traditionally classified into the spotted fever group and the typhus group, encompassing several pathogenic species responsible for human disease worldwide. Transmission occurs through arthropod vectors such as ticks, fleas, mites, and lice, with tick-borne rickettsioses representing the most common forms reported in Mexico [1-4].

Rickettsial infections remain an important public health concern in Mexico, particularly in northern border states where the highest incidence and mortality have been reported. Recent analyses identified 4,373 reported cases of Rocky Mountain spotted fever and other spotted fever group rickettsioses in five northern Mexican border states between 2009 and 2023, highlighting the persistent burden of these infections in the country [4].

The clinical manifestations of rickettsial infections range from mild self-limited febrile illnesses to severe multisystem disease. Classically, patients present with fever, headache, and rash, frequently accompanied by epidemiological exposure to arthropod vectors. However, delayed diagnosis is common because early symptoms are nonspecific and may mimic viral or other bacterial infections. In severe cases, endothelial invasion and widespread vasculitis can lead to thrombocytopenia, acute kidney injury, hepatic dysfunction, shock, and multiple organ failure [2-4].

Neurological involvement is an uncommon but potentially devastating complication of rickettsial infections. Reported manifestations include encephalopathy, meningitis, meningoencephalitis, seizures, focal neurological deficits, and cerebral vasculitis. Central nervous system involvement has been associated with increased mortality and a higher risk of long-term neurological sequelae, particularly when initiation of doxycycline is delayed [5-13].

Diagnosis relies primarily on clinical suspicion supported by epidemiological risk factors and laboratory findings. Molecular techniques such as polymerase chain reaction (PCR) may facilitate early diagnosis during the acute phase of illness, whereas indirect immunofluorescence assay (IFA) remains the reference serologic method. Because outcomes are strongly influenced by the timing of treatment, current recommendations emphasize prompt initiation of doxycycline whenever rickettsiosis is suspected, even before microbiological confirmation is obtained [14].

We report the case of a previously healthy young adult who developed severe meningoencephalitis associated with *Rickettsia *spp. infection, complicated by multiorgan failure and persistent neurological deterioration despite intensive care support and targeted antimicrobial therapy. This case highlights the importance of early recognition of rickettsiosis in patients presenting with fever, rash, and acute neurological symptoms in endemic regions.

## Case presentation

A 28-year-old man with no significant past medical history presented with a six-day history of febrile illness and progressive neurological deterioration. His epidemiological history was notable for close contact with a vaccinated dog and direct exposure to ticks. He reported social alcohol consumption and had an unknown immunization status.

The illness began with non-specific symptoms consisting of fever and acute gastroenteritis characterized by approximately four bowel movements per day, for which he received empirical treatment with ceftriaxone. Despite treatment, on the fourth day of illness, he developed an erythematous macule on the lateral aspect of the left leg, which progressed to a maculopapular lesion and subsequently evolved into a solitary, flat, hyperpigmented gray-black lesion measuring approximately 3-4 cm in diameter with irregular but well-defined borders (Figure 1). During the same period, he developed an erythematous macular rash involving the trunk with centrifugal progression to the extremities, including the palms and soles (Figures 2-4). Concurrently, he experienced progressive neurological symptoms characterized by somnolence, disorganized speech, reduced verbal output, and impaired comprehension.

**Figure 1:**
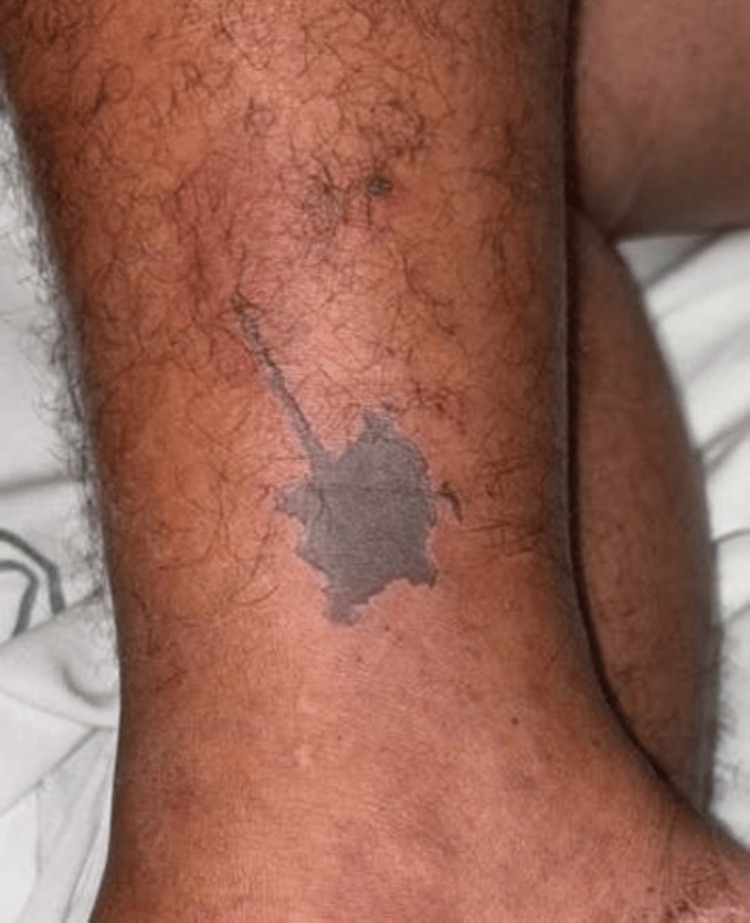
Inoculation eschar on the left leg. A cutaneous lesion was located on the lateral aspect of the left leg, characterized by a central necrotic-appearing area surrounded by an erythematous halo. The lesion was subsequently recognized as an inoculation eschar, supporting the diagnosis of rickettsial infection. It measured approximately 3 × 4 cm at its greatest dimensions.

**Figure 2:**
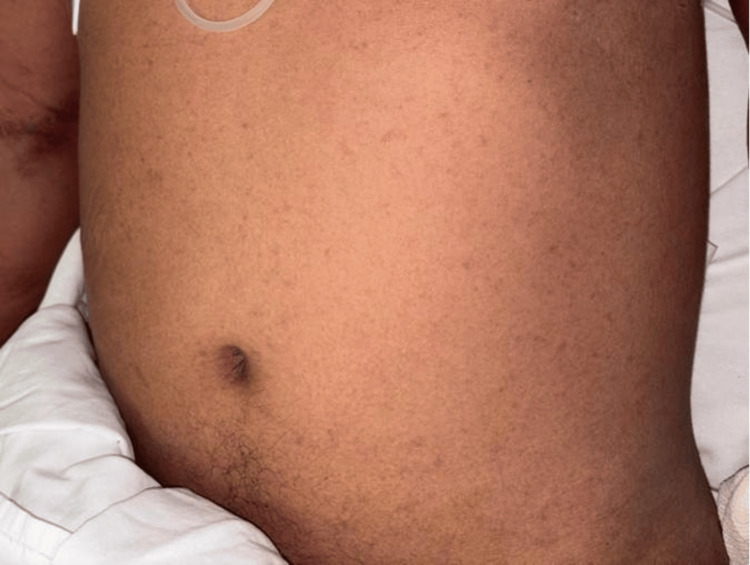
Macular exanthem involving the abdomen. Multiple erythematous macules were diffusely distributed over the anterior trunk, representing the characteristic exanthem observed during the acute presentation.

**Figure 3:**
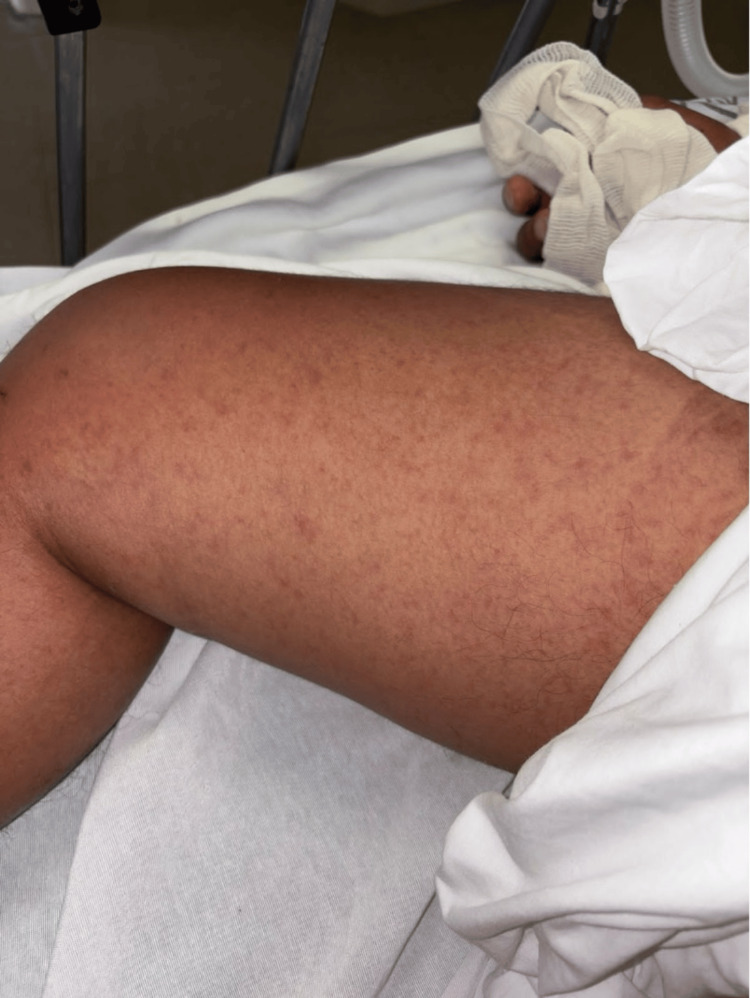
Lower extremity involvement by the exanthem. Numerous discrete erythematous macules were distributed throughout the thigh.

**Figure 4:**
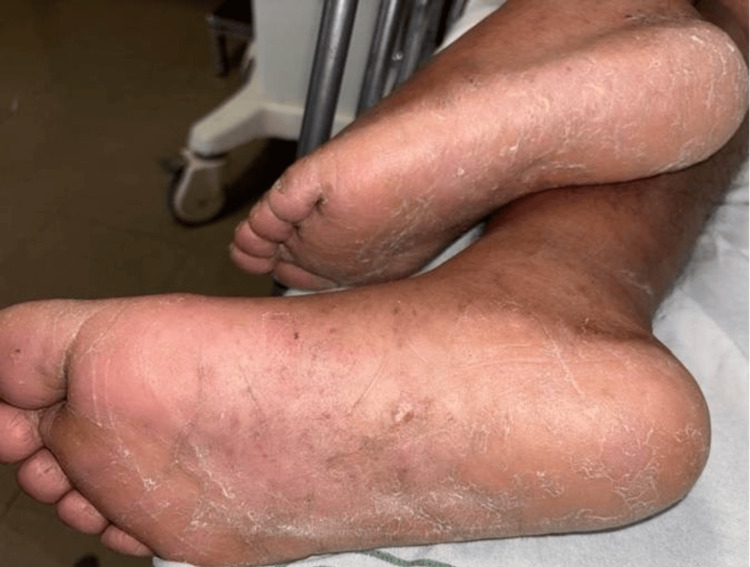
Plantar surfaces showing scattered erythematous macules with associated superficial desquamation.

He was admitted on the sixth day of illness with a temperature of 38°C and severe neurological impairment, progressing to stupor and requiring endotracheal intubation and mechanical ventilation for airway protection. Initial laboratory evaluation revealed leukocytosis with neutrophilia, severe thrombocytopenia, acute kidney injury, hyponatremia, elevated liver enzymes, and hyperbilirubinemia (Table 1).

**Table 1:** Serial laboratory findings during hospitalization. Laboratory evaluation demonstrated leukocytosis, severe thrombocytopenia, acute kidney injury, electrolyte abnormalities, and hepatic dysfunction at admission, with dynamic changes observed throughout hospitalization. LDH: lactate dehydrogenase; AST: aspartate transaminase; ALT: alanine transaminase; INR: International Normalized Ratio; pCO_2_: partial pressure of carbon dioxide; pO_2_: partial pressure of oxygen; NR: not recorded

Parameter	Reference Range	Admission Day 1	Hospital Day 6	Hospital Day 10
Hemoglobin (g/dL)	13.5-17.5	13.2	8.8	10.7
Hematocrit (%)	41-53	36.5	27.0	33.6
Platelet count (cells/µL)	150,000-450,000	45,000	244,000	393,000
Leukocyte count (cells/mm³)	4,000-10,000	16,000	10,900	15,200
Lymphocyte count (cells/mm³)	1,000-4,800	1,500	1,800	2,400
Neutrophil count (cells/mm³)	1,500-7,500	13,100	8,100	11,200
Prothrombin time (sec)	10-13	10.4	10.0	13.5
INR	0.8-1.2	0.93	0.90	1.10
Activated partial thromboplastin time (sec)	25-35	30.3	23.6	29.4
Glucose (mg/dL)	70-100	122	110	107
Urea (mg/dL)	15-40	181	69	264
Blood urea nitrogen (mg/dL)	7-20	84.4	32.0	123.2
Creatinine (mg/dL)	0.7-1.3	3.47	0.73	8.03
Chloride (mmol/L)	98-107	94.3	119.0	101.0
Sodium (mmol/L)	135-145	126.6	154.4	139.8
Potassium (mmol/L)	3.5-5.1	4.24	3.68	6.69
Magnesium (mg/dL)	1.7-2.4	2.85	NR	2.47
Calcium (mg/dL)	8.5-10.5	7.81	NR	7.10
Phosphorus (mg/dL)	2.5-4.5	5.7	NR	NR
AST (U/L)	10-40	147	63.9	1440
ALT (U/L)	7-56	81.4	84.4	1427
Total bilirubin (mg/dL)	0.2-1.2	2.59	0.39	3.56
Direct bilirubin (mg/dL)	0.0-0.3	1.90	NR	2.52
LDH (U/L)	140-280	555	236	NR
Total protein (g/dL)	6.0-8.3	4.9	NR	NR
Albumin (g/dL)	3.5-5.0	2.7	NR	NR
pH	7.35-7.45	7.39	7.41	7.23
pCO₂ (mmHg)	35-45	31	43	38
pO₂ (mmHg)	80-100	53	51	121
Lactate (mmol/L)	0.5-2.0	1.5	0.8	2.4
Bicarbonate (mmol/L)	22-28	20.6	26.5	16.4
Base excess (mmol/L)	-2 to +2	-6.2	2.3	-10.9
Oxygen saturation (%)	95-100	87	86	98
C-reactive protein (mg/dL)	<0.5	71.3	NR	NR
Amylase (U/L)	30-110	NR	NR	436
Lipase (U/L)	10-140	NR	NR	342

Non-contrast computed tomography (CT) of the brain was performed following neurological deterioration. Imaging demonstrated subtle loss of gray-white matter differentiation and poor visualization of cortical sulci and gyri, with mild sulcal effacement, findings suggestive of diffuse cerebral edema (Figures 5-6). No intracranial hemorrhage, mass effect, or midline shift was identified.

**Figure 5:**
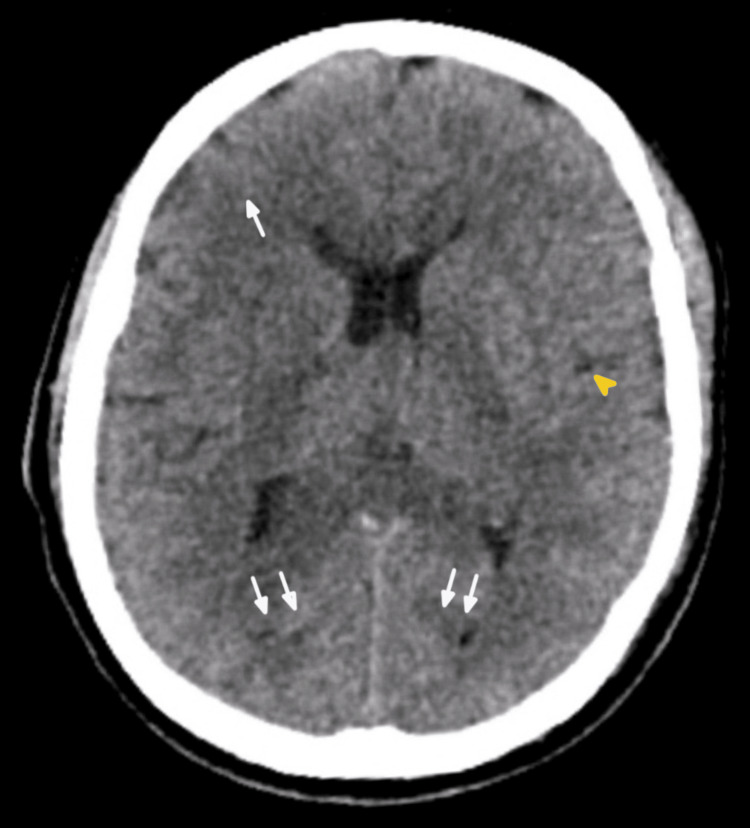
Non-contrast brain CT at the level of the basal ganglia. Axial non-contrast CT image demonstrating mild loss of gray-white matter differentiation (white arrows) and effacement of the Sylvian fissure (yellow arrowhead). These findings were suggestive of diffuse cerebral edema. No intracranial hemorrhage, focal ischemic lesions, mass effect, or midline shift was identified. CT: computed tomography

**Figure 6:**
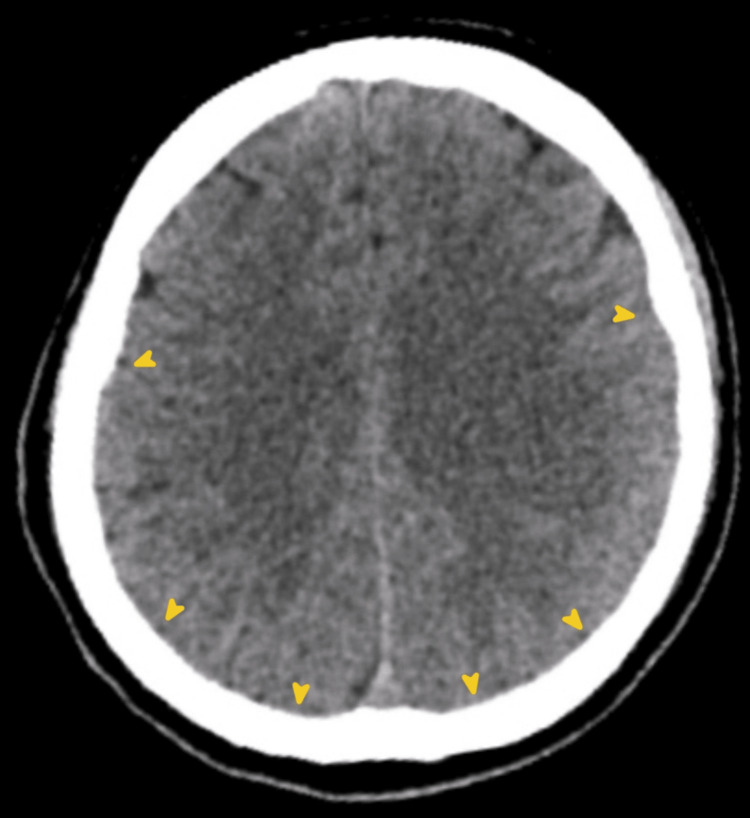
Non-contrast brain CT at a high supratentorial level. Axial non-contrast CT image demonstrating mild diffuse cerebral edema (yellow arrowheads) with slight loss of normal sulcal prominence. No focal intracranial hemorrhage, space-occupying lesion, or significant midline shift was observed. CT: computed tomography

Cerebrospinal fluid analysis revealed 20 leukocytes/mm³ (50% polymorphonuclear cells and 50% mononuclear cells), a protein concentration of 92 mg/dL, and glucose of 68 mg/dL. A multiplex meningitis/encephalitis PCR panel (BioFire® FilmArray®; BioFire Diagnostics, LLC, Salt Lake City, USA) was negative for common bacterial, viral, and fungal pathogens. Blood cultures remained sterile. Conventional PCR testing performed on a serum sample was positive for *Rickettsia *spp. The assay confirmed the presence of *Rickettsia *DNA but did not provide species-level identification; therefore, the causative species could not be determined.

A focused re-examination identified the lesion on the left leg as an inoculation eschar, further supporting the diagnosis of rickettsial infection. Empirical treatment for suspected central nervous system infection had been initiated with intravenous vancomycin (2 g loading dose followed by 1 g every eight hours), ceftriaxone (2 g every 12 hours), acyclovir (700 mg every eight hours), and dexamethasone (10 mg every six hours). Following the positive PCR result and the epidemiological and clinical findings suggestive of rickettsiosis, intravenous doxycycline 200 mg once daily was added on day 6 of illness (hospital day 2).

The patient received intensive supportive care, including mechanical ventilation, correction of electrolyte disturbances, and supportive management of organ dysfunction. Follow-up laboratory studies initially showed partial improvement in selected abnormalities, including platelet count and some electrolyte parameters. However, the patient subsequently developed marked worsening of renal and hepatic function, with rising creatinine and transaminase levels, consistent with progressive multiorgan failure. Despite intensive supportive care and targeted antimicrobial therapy, no meaningful neurological recovery was observed.

During hospitalization, the patient developed progressive hemodynamic instability requiring vasopressor support. His clinical course was further complicated by multiorgan failure involving the hepatic, pancreatic, renal, and pulmonary systems. Despite targeted antimicrobial therapy and intensive care management, neurological recovery was not achieved after withdrawal of sedation. Neuroimaging findings suggestive of diffuse cerebral edema further supported central nervous system involvement. The patient continued to deteriorate despite maximal supportive measures and ultimately died during hospitalization as a consequence of progressive multiorgan failure.

## Discussion

Rickettsioses are systemic infectious diseases that frequently present with nonspecific symptoms during the early stages of illness, making initial diagnosis challenging and often leading to confusion with viral or other bacterial infections [1-3]. Neurological involvement is an uncommon but potentially devastating complication associated with increased morbidity and mortality. Several reports have described encephalitis and meningoencephalitis secondary to spotted fever group rickettsioses, including cases caused by *Rickettsia conorii*, severe neuromeningeal involvement associated with spotted fever group rickettsiae, and central nervous system infection caused by *Rickettsia felis* complicated by cerebrovascular events [5,7-13].

The pathogenesis of severe rickettsial disease is largely explained by the organism’s tropism for vascular endothelial cells, resulting in diffuse vasculitis, endothelial injury, increased vascular permeability, and tissue ischemia [1-3]. This mechanism accounts for many of the clinical manifestations observed in severe disease, including thrombocytopenia, acute kidney injury, hepatic dysfunction, shock, and multiorgan failure [1-3]. Recent reports have also documented cerebral vasculitis with vascular enhancement and multifocal ischemic lesions on neuroimaging, supporting endothelial injury as a major contributor to neurological complications [11,12].

In the present case, neurological manifestations developed early in the disease course and progressed rapidly to severe encephalopathy requiring mechanical ventilation. Several alternative contributors to neurological deterioration were considered, including metabolic encephalopathy secondary to acute kidney injury, uremia, hyponatremia, hepatic dysfunction, and, later in the clinical course, multiorgan failure. Each of these abnormalities has been associated with altered mental status and could have contributed to the patient's neurological presentation.

However, several findings supported a primary infectious neurological process. Neurological symptoms emerged during the acute febrile illness and were accompanied by a characteristic exanthem, thrombocytopenia, and epidemiological exposure to ticks. Cerebrospinal fluid analysis demonstrated inflammatory abnormalities, including pleocytosis and elevated protein concentration, findings that are not typically explained by metabolic encephalopathy alone. Furthermore, conventional PCR testing confirmed infection with* Rickettsia *spp., and the patient exhibited additional features highly suggestive of rickettsial disease, including fever, a centrifugal rash involving the palms and soles, and the presence of an inoculation eschar. Although some electrolyte abnormalities and thrombocytopenia initially improved, neurological recovery was not observed, and the patient subsequently developed worsening renal and hepatic dysfunction consistent with progressive multiorgan failure. Taken together, these findings support rickettsial meningoencephalitis as the principal cause of neurological deterioration, while acknowledging that metabolic derangements likely contributed to the overall severity of the clinical presentation.

The diagnosis of rickettsiosis remains largely dependent on clinical suspicion. Molecular techniques such as PCR may facilitate early diagnosis during the acute phase of illness, whereas indirect IFA remains the reference standard for serological confirmation [14]. However, because laboratory confirmation may not be immediately available, current recommendations emphasize that treatment should not be delayed when rickettsiosis is clinically suspected [14].

An important limitation of this case is that the diagnosis was established by conventional PCR, which confirmed infection with *Rickettsia *spp. but did not provide species-level characterization. Therefore, the specific rickettsial species responsible for the infection could not be determined. References to individual species reported in the literature are included solely to illustrate the spectrum of neurological manifestations associated with rickettsial infections and should not be interpreted as evidence that the present case was caused by a particular species.

Another limitation is that direct central nervous system involvement could not be microbiologically confirmed because PCR testing was performed on serum rather than cerebrospinal fluid. Furthermore, neuroimaging did not demonstrate specific inflammatory lesions. Consequently, the diagnosis of rickettsial meningoencephalitis was based on the combination of compatible neurological manifestations, cerebrospinal fluid inflammatory findings, epidemiological exposure, characteristic dermatological manifestations, exclusion of alternative etiologies, and positive serum PCR for *Rickettsia *spp.

Doxycycline remains the treatment of choice for all suspected rickettsial infections, regardless of patient age, with standard therapy consisting of 100 mg twice daily for at least seven days or until clinical improvement is achieved [14]. Multiple case series and reports of rickettsial encephalitis have demonstrated that delayed initiation of doxycycline is associated with worse neurological outcomes, increased mortality, and a greater risk of permanent neurological sequelae [7,10-13].

Several factors associated with severe disease and poor outcomes described in the literature were present in our patient, including severe thrombocytopenia, acute kidney injury, hepatic dysfunction, shock requiring vasopressor support, mechanical ventilation, and subsequent multiorgan failure [7,13]. Although supportive care resulted in transient improvement of selected laboratory abnormalities, renal and hepatic function subsequently deteriorated, reflecting progressive systemic involvement. Neurological recovery was not observed despite intensive care management and targeted antimicrobial therapy. The delayed recognition of the underlying etiology likely contributed to the unfavorable outcome.

This case highlights the diagnostic challenge posed by rickettsial infections presenting with predominant neurological manifestations. In endemic regions, rickettsiosis should be considered in patients presenting with fever, rash, and acute neurological deterioration, particularly when accompanied by thrombocytopenia, hyponatremia, acute kidney injury, or epidemiological exposure to ticks. Although definitive laboratory confirmation may not be immediately available, early clinical recognition and prompt initiation of doxycycline remain critical to reducing morbidity and mortality. Increased awareness of atypical neurological presentations may facilitate earlier diagnosis and improve patient outcomes.

## Conclusions

This case highlights the potential severity of rickettsial infection and its ability to initially present with nonspecific symptoms, delaying recognition of a rapidly progressive systemic disease. Neurological manifestations may occur early in the clinical course and can be associated with multiorgan dysfunction and poor outcomes. Although direct microbiological confirmation of central nervous system infection was not available, the combination of acute neurological deterioration, cerebrospinal fluid inflammatory abnormalities, characteristic cutaneous findings, epidemiological exposure to ticks, and positive serum PCR for *Rickettsia *spp. strongly supported rickettsial meningoencephalitis as the most likely diagnosis. In endemic regions, rickettsiosis should be included in the differential diagnosis of patients presenting with fever, rash, and acute neurological deterioration, particularly when accompanied by thrombocytopenia, acute kidney injury, or relevant epidemiological risk factors. Early clinical suspicion and prompt initiation of doxycycline, without waiting for laboratory confirmation, remain essential to improve outcomes, reduce mortality, and prevent irreversible neurological complications.
